# RNA binding protein HuD regulates fatty acid oxidation in pancreatic β-cells by modulating long-chain acyl-CoA dehydrogenase expression

**DOI:** 10.1080/19768354.2025.2542168

**Published:** 2025-08-11

**Authors:** Jiyoon Seo, Seungyeon Ryu, Wei Zhang, Eun Kyung Lee, Seung Min Jeong

**Affiliations:** a Department of Biochemistry, College of Medicine, The Catholic University of Korea, Seoul, Republic of Korea; b Department of Medical Science, Graduate School of The Catholic University of Korea, Seoul, Republic of Korea; c Shanghai Public Health Clinical Center, Shanghai Medical College, Fudan University, Shanghai, People’s Republic of China; d Institute for Aging and Metabolic Diseases, College of Medicine, The Catholic University of Korea, Seoul, Republic of Korea

**Keywords:** HuD, LCAD, fatty acid oxidation, lipotoxicity, pancreatic β-cell

## Abstract

RNA binding proteins (RBPs) play crucial roles in the post-transcriptional regulation of metabolic pathways. Although the RBP HuD has been extensively studied in pancreatic β-cells, its role in cellular metabolism remains poorly understood. In this study, we uncover a novel function of HuD in regulating fatty acid oxidation (FAO) in mouse insulinoma βTC6 cells. Through genetic knockdown and overexpression approaches, we demonstrate that HuD modulates the expression of long-chain acyl-CoA dehydrogenase (LCAD), a key enzyme in FAO, by binding to the 3′-untranslated region of its mRNA. Loss of HuD impaired FAO, leading to lipid droplet accumulation, elevated reactive oxygen species production, and increased lipotoxicity under lipid-stress conditions. These findings reveal a previously unrecognized role for HuD in maintaining fatty acid homeostasis and suggest that the HuD-LCAD regulatory axis may represent a promising therapeutic target for preserving β-cell integrity and function.

## Introduction

Fatty acid metabolism plays a crucial role in maintaining cellular energy balance and overall metabolic homeostasis (Wakil and Abu-Elheiga 2009). Fatty acids serve as essential energy substrates, particularly in tissues with high energy demands, such as the heart, liver, and pancreatic β-cells (Smith et al. 2018). However, dysregulation of fatty acid metabolism can lead harmful effects to cells. For example, excess fatty acids can act as acids by releasing protons (H^+^), leading to intracellular acidification that disrupts enzymatic activity and mitochondrial function. This acidification, combined with the accumulation of free fatty acids (FFAs), increases oxidative stress by overloading the electron transport chain and promoting excessive reactive oxygen species (ROS) production (Wajner and Amaral 2015). Elevated ROS levels cause mitochondrial dysfunction, lipid peroxidation, and DNA damage, further exacerbating cellular stress. Additionally, FFAs contribute to lipotoxicity by generating toxic lipid intermediates, such as ceramides and diacylglycerols, which trigger endoplasmic reticulum stress, inflammation, and apoptosis (Oh et al. 2018; Yoon et al. 2021). This combination of acidification, ROS generation, and toxic lipid accumulation severely impairs cellular function, particularly in metabolically active cells like pancreatic β-cells (Vianey-Saban et al. 2023), highlighting the importance of maintaining lipid homeostasis to prevent lipotoxic damage.

Fatty acid oxidation (FAO), especially long-chain fatty acid (LCFA) β-oxidation, is a vital metabolic process that provides energy by breaking down fatty acids into acetyl-CoA, which subsequently enters the tricarboxylic acid (TCA) cycle for ATP production (Houten et al. 2016). Moreover, FAO plays a pivotal role in maintaining fatty acid homeostasis by breaking down excess FFAs and preventing toxic lipid accumulation. FAO occurs in the mitochondria through a series of steps, including activation of fatty acids to acyl-CoA, transport into the mitochondrial matrix via the carnitine shuttle, and sequential β-oxidation cycles that shorten the fatty acid chain by two carbons per cycle (Vianey-Saban et al. 2023). A crucial group of enzymes in this process are the acyl-CoA dehydrogenases (ACADs), which catalyze the initial dehydrogenation step of β-oxidation, transferring electrons to the electron transfer flavoprotein (ETF) (Swigonova et al. 2009). These enzymes are classified based on their substrate specificity, ranging from short- to long-chain acyl-CoAs. Normally, fatty acid usage and synthesis are tightly regulated to avoid intra cellular lipid accumulation. In particular, mutations in ACAD genes are associated with metabolic disorders, which result in impaired FAO, leading to severe energy deficits, hypoglycemia, and lipid accumulation (Houten et al. 2016). Among these enzymes, long-chain acyl-CoA dehydrogenase (LCAD) plays a particularly significant role in β-oxidation of long-chain fatty acids, which are the predominant lipid species metabolized by many tissues (Kurtz et al. 1998; Zhang et al. 2019). However, the regulatory mechanisms of LCAD remain poorly understood, emphasizing the need for further research to elucidate its role in lipid metabolism and energy homeostasis.

RNA-binding proteins (RBPs) play a fundamental role in post-transcriptional gene regulation, influencing mRNA stability, localization, and translation (Hentze et al. 2018). These proteins are essential for numerous cellular processes, including metabolism, differentiation, and stress responses (Gebauer et al. 2021). Among them, the RBP HuD, a member of the ELAV (embryonic lethal abnormal vision)-like protein family, is predominantly expressed in neurons and pancreatic β-cells (Jung and Lee 2021 apr 23). In β-cells, HuD has been implicated in regulating insulin synthesis, secretion, and cell survival by stabilizing key mRNAs involved in β-cell function (Hong et al. 2020; Jung et al. 2022 dec 5; Cha et al. 2024). While roles of HuD in neuronal development and β-cell physiology is well established, its function in metabolic regulation remains largely unexplored. Recent evidence suggests a link between HuD and lipid metabolism in pancreatic β-cells. HuD has been shown to enhance triglyceride production by stabilizing mRNA of insulin-induced gene 1 (Insig1), a critical repressor of lipogenesis, thereby implicating its role in the regulation of lipid storage (Kim et al. 2016). However, the role of HuD in lipid catabolism, particularly FAO, remains unexplored.

Here, we identify a novel function of HuD in regulating FAO in pancreatic β-cells. Our findings demonstrate that downregulation of HuD impairs FAO by decreasing the expression of LCAD. This reduction in LCAD expression leads to diminished β-oxidation of long-chain fatty acids, resulting in lipid accumulation and heightened cellular stress. These results underscore the essential role of HuD in maintaining fatty acid homeostasis in β-cells through the regulation of FAO, thereby preventing lipotoxicity and supporting β-cell function under conditions of lipid stress.

## Materials & methods

### Cell culture and transfection

The mouse pancreatic β-cell line βTC6 was cultured in Dulbecco's Modified Eagle Medium (DMEM) high glucose (WelGENE, South Korea) supplemented with 10% fetal bovine serum (Gibco, USA) and penicillin/streptomycin (Biowest, France). βTC6 cell clones stably expressing shHuD plasmid (Santa Cruz Biotechnology, USA) (βTC6-shHuD) were established and maintained with puromycin. The HuD overexpression plasmid was generated by cloning CDS of mouse HuD mRNA into a retroviral vector pPGS-HA and viral particles were prepared using HEK293 cells. An enhanced green fluorescent protein (EGFP) reporter construct was generated by cloning the 3'UTR of LCAD mRNA (NM_007381.4, 1354-1948nt) downstream of the EGFP coding sequence in the pEGFP-C1 vector (BD Biosciences, USA). Transfection of small interfering RNAs (control siRNA, siCon; HuD siRNA, siHuD) (Genolution Pharmaceuticals, South Korea) or plasmids was performed using Lipofectamine™ 2000 (Invitrogen™, USA) according to the manufacturer’s instructions.

### Western blot analysis

Cells were lysed in RIPA lysis buffer (EzRIPA lysis kit, ATTO, Japan) supplemented with protease and phosphatase inhibitor cocktails. Protein lysates were separated by sodium dodecyl sulfate-polyacrylamide gel electrophoresis and transferred onto polyvinylidene fluoride membranes. Membranes were blocked with 3% bovine serum albumin and incubated with primary antibodies overnight at 4°C. After four washes with 1X TBST, membranes were incubated with species-specific secondary antibodies and detected using the LAS 4000 (Fuji film, Japan) imaging system. To assess de novo protein synthesis, cells were incubated in methionine-free medium for 1 h, followed by treatment with L-azidohomoalanine (AHA)-containing medium for 4 h to allow incorporation of the methionine analog. Nascent proteins were biotinylated using a Click-iT™ reaction buffer (Invitrogen™) and enriched with streptavidin-conjugated Dynabeads (Invitrogen™). The isolated proteins were analyzed by Western blotting using an LCAD antibody.

### ROS determination

Cells were seeded and incubated overnight. The following day, cells were treated with or without palmitate (Sigma-Aldrich, USA) for 24 h. Cellular and mitochondrial superoxide levels were assessed using DCFDA (Invitrogen™) according to the manufacturer’s instructions. Cells were washed with phosphate-buffered saline (PBS) containing 10 μM DCFDA, trypsinized, and resuspended in Hank's balanced salt solution (HBSS). Fluorescence intensity was measured by flow cytometry using a FACSCanto system (BD Biosciences, USA).

### Quantitative RT–PCR

Total RNA was prepared with TRIzol reagent (Invitrogen™) according to the manufacturer’s instructions. 1 μg of RNA was reverse-transcribed using the iScript cDNA synthesis kit (Bio-Rad, USA). Diluted cDNA samples were analyzed by real-time PCR using SYBR Green I Master Mix on a LightCycler 480 system (Roche, USA). Gene expression levels were normalized to β-Actin. Primer sequences were as follows:

GGCTCTCCAAGGCTGTATG and ACCACTGCGACTTAACTCTG for mouse VLCAD; TTTCCTCGGAGCATGACATTTT and GCCAGCTTTTTCCCAGACCT for mouse LCAD; TCGGTGAAGGAGCAGGTTTCAAGA and AAACTCCTTGGTGCTCCACTAGCA for mouse MCAD; GAGCTTGGCTGCCTCTTTAC and CATGGGAACAGCACTGAGAG for mouse SCAD; AGCCATGTACGTAGCCATCC and CTCTCAGCTGTGGTGGTGAA for mouse β-Actin

### Antibodies

The following antibodies were used: β-Actin (Genetex, USA, GTX109639), VLCAD (Proteintech, USA, 14527-1-AP), LCAD (Proteintech, 17526-1-AP), MCAD (Abcam, UK, ab92461), SCAD (Abcam, ab156571), HuD (Santa Cruz, sc-28299), PARP1 (Abcam, ab32138), mouse IgG (Santa Cruz, sc-2025)

### Cell death assay

Cells were seeded and incubated overnight before treatment with or without palmitate for 24 h. After treatment, cells were trypsinized, centrifuged, washed with PBS and resuspended in PBS. Cell death was evaluated by flow cytometry using propidium iodide (PI, 0.5 μg/ml) staining.

### RNA immunoprecipitation

Ribonucleoprotein (RNP) complexes were immunoprecipitated from cell lysates using Protein A beads conjugated with HuD or control IgG antibodies. RNA was isolated from the complexes through sequential DNase I and Proteinase K digestion. The enriched RNA was reverse transcribed to cDNA and analyzed by qPCR using gene-specific primers. Data were processed using the ΔΔC_T_ method to compare control and experimental groups. GAPDH mRNA was used for normalization. Primer sequence were as follows: AGGTCGGTGTGAACGGATTTG and TGTAGACCATGTAGTTGAGGTCA for mouse GAPDH.

### Biotin pull-down assay

DNA fragments corresponding to the 5'UTR and 3'UTR of LCAD mRNA (NM_007381.4) were amplified by PCR using forward primers containing a T7 RNA polymerase binding site (5′-CCAAGCTTCTAATACGACTCACTATAGGGAGA-3′). Primers sequences were: CCAAGCTTCTAATACGACTCACTATAGGGAGATCTGTCCGCCTGCCCCCGCCGCGGA and GGCGGGATGGACGGCGGCGGACG for mouse LCAD 5’UTR; CCAAGCTTCTAATACGACTCACTATAGGGAGAACATCTGCCTACATCCTGGAAT and CACGGTCTACACATGCTTTAAGT for mouse LCAD 3’UTR. Biotinylated transcripts were synthesized using the MaxiScript T7 kit (Ambion, USA) with biotin-CTP (Enzo Life Sciences, USA). Transcripts were purified using the MEGAclear™ Kit (Invitrogen™) and incubated with whole-cell lysates for 30 min at room temperature. Biotinylated RNA-protein complexes were precipitated using streptavidin-coated Dynabeads (Invitrogen™). Proteins were detected via Western blotting using a HuD antibody.

### Cell staining and microscopy

A stock solution of Oil Red O (Sigma-Aldrich) was prepared by dissolving powder in isopropanol. A working solution was prepared by mixing the stock solution and ddH₂O and filtered through a 0.45 μm filter. Cells were fixed with 4% paraformaldehyde, washed with 60% isopropanol, and stained with Oil Red O working solution at room temperature. Stained cells were observed using an IX70 microscope (Olympus Corp., Japan) and quantification was performed by measuring absorbance. For lipid droplet staining, cells were incubated with 0.2 μM Nile Red and counterstained with DAPI (Invitrogen™). Fluorescence images were observed using a ZEISS Axio Observer. Z1 Inverted Fluorescence microscope (Carl Zeiss, Germany), and lipid droplet quantification was performed using ImageJ. A stock solution of FAOBlue (Funakoshi, Japan, FDV-0033) was prepared by dissolving the powder in DMSO. A working solution was prepared by diluting the stock solution in HEPES-buffered saline (HBS). For FAOBlue staining, cells were incubated with 10 μM FAOBlue diluted in HBS for 1 h at 37°C. Fluorescence intensity was measured using microplate reader and visualized using LSM 8000 (Carl Zeiss). To normalize fluorescence intensity, cell viability was assessed using the Cell Counting Kit-8 (Dojindo, Japan) according to the manufacturer’s instructions.

### Statistical analysis

Data were presented as mean ± SD from three independent experiments. Statistical significance of data was determined using Student’s t-test or two-way ANOVA with Tukey’s, Dunnett’s or Sidak’s multiple comparisons test. (*p < 0.05; **p < 0.01; ***p < 0.001; ****p < 0.0001.)

## Results

### Depletion of HuD suppresses fatty acid oxidation and induces lipid accumulation in pancreatic β-cells

**Figure 1.**
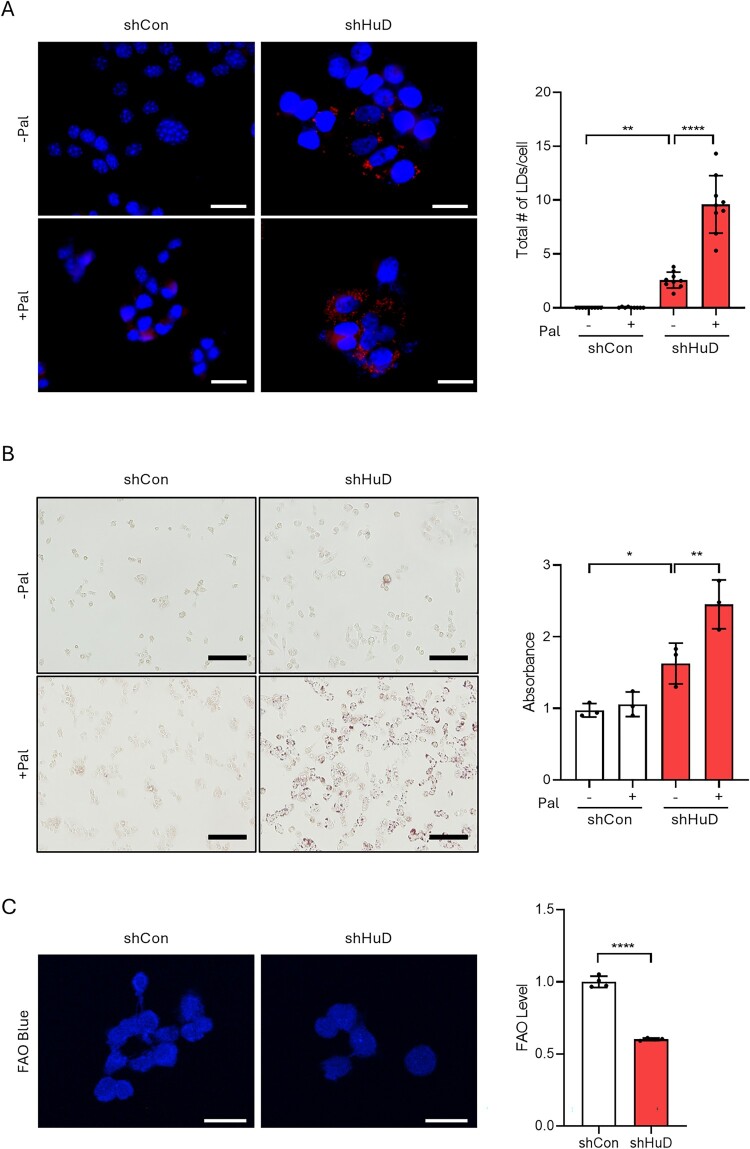
HuD downregulation leads to FAO impairment and lipid accumulation in pancreatic β-cells. (A,B) shCon and shHuD cells were treated with or without palmitate. (A) Cells were stained with Nile Red to visualize lipid droplets and counterstained with DAPI. Red fluorescence intensity was quantified using ImageJ software. Scale bar represents 20 μm. Statistical significance was determined using two-way ANOVA followed by Tukey’s multiple comparisons test. (B) Cells were stained with Oil-Red-O solution and levels of staining was quantified by measuring absorbance at 510 nm. Scale bar represents 100 μm. Statistical significance was determined using a two-way ANOVA with Tukey’s multiple comparisons test. (C) Cells were stained with FAOBlue and levels of staining was quantified by measuring fluorescence. Scale bar represents 20 μm. Statistical significance was determined using Student’s t-test. Representative images are shown and data are presented as mean ± SD from each image. (*p < 0.05; **p < 0.01; ****p < 0.0001.)

Cellular fatty acid homeostasis is essential for proper pancreatic β-cell function (Han et al. 2021 mar). To investigate the role of HuD in fatty acid metabolism, we generated HuD knockdown mouse insulinoma βTC6 cells, in which HuD expression was stably reduced using retroviral shRNA targeting HuD (Supplementary Figure S1A). Consistent with previous findings (Kim et al. 2016), HuD knockdown cells exhibited slightly increased lipid accumulation, as demonstrated by enhanced fluorescence intensity in Nile Red staining and elevated lipid deposition in Oil Red O staining (Figure 1A, B). Notably, whereas supplementation of palmitate – a predominant circulating lipotoxic saturated FFA that is elevated in obese individuals – had no significant effect on lipid accumulation in control cells, it markedly exacerbated lipid accumulation in HuD-depleted cells (Figure 1A, B). These findings suggest that HuD may play a critical role in maintaining fatty acid homeostasis in pancreatic β-cells.

Defects in mitochondrial FAO can lead to intracellular lipid accumulation and the reduced expression of FAO-related genes was associated with enhanced lipid accumulation in various cells (Kang et al. 2015; Acosta-Montano and Garcia-Gonzalez 2018; Zeng et al. 2022). Thus, we hypothesized that HuD regulates mitochondrial FAO. To examine the capacity alteration of FAO by HuD depletion, we examined FAO capacity using FAO-blue dye. When we measured the intensity of fluorescence by confocal microscopy, HuD knockdown cells exhibited a significant reduction in FAO (Figure 1C). Taken together, these findings demonstrate that HuD depletion disrupts FAO, leading to dysregulation in FA homeostasis under lipid-overloaded conditions.

### LCAD is a downstream target of HuD in pancreatic β-cells

**Figure 2.**
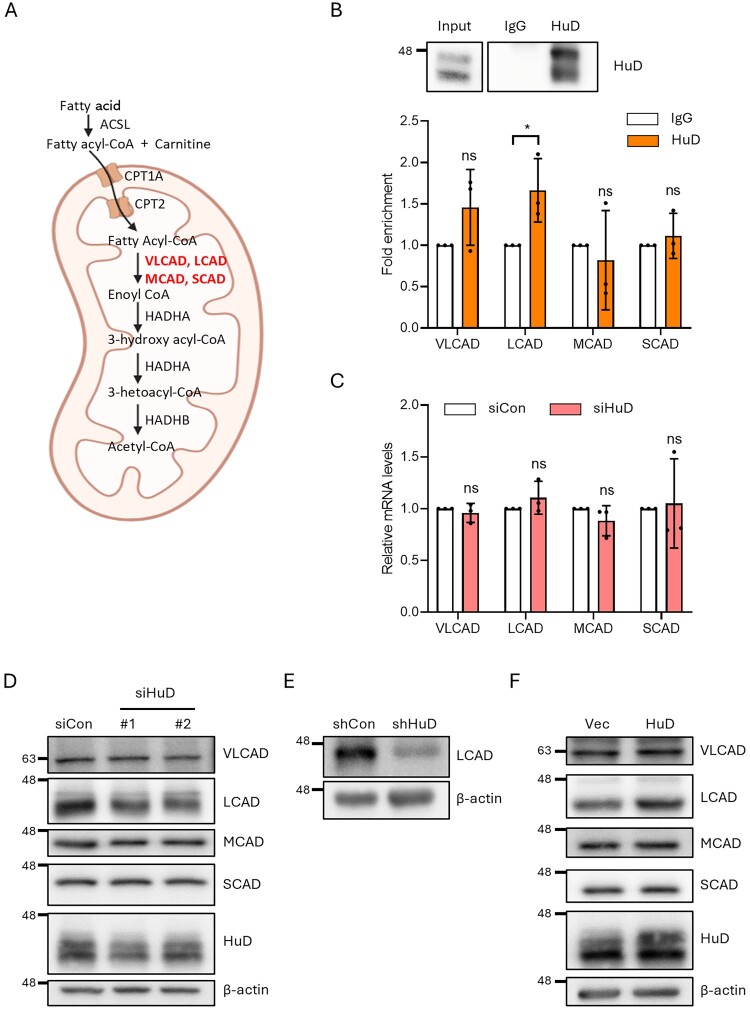
HuD modulates the expression of LCAD in pancreatic β-cells. (A) Schematic representation of mitochondrial fatty acid oxidation. (B) Interaction between HuD and putative target mRNAs encoding chain acyl-CoA dehydrogenases was analyzed by RNA immunoprecipitation using HuD or control IgG antibodies, followed by RT-qPCR. Statistical significance was determined using Student’s t-test. (C) Relative expression levels of chain acyl-CoA dehydrogenase genes in βTC6 cells. Statistical significance was determined using two-way ANOVA followed by Sidak’s multiple comparisons test. (D) Protein levels of VLCAD, LCAD, MCAD, SCAD and HuD in whole-cell lysates from βTC6 cells transfected with either control siRNA (siCon) or two independent siRNAs targeting HuD (siHuD). (E) LCAD protein levels in whole-cell lysates from βTC6 cells transfected with either control shRNA (shCon) or shRNA targeting HuD (shHuD). Protein levels of VLCAD, LCAD, MCAD, SCAD and HuD in whole-cell lysates from βTC6 cells transfected with either an empty vector (Vec) or HuD overexpression construct (HuD). (F) Representative images are shown and data are presented as mean ± SD from each image. (*p < 0.05; ns, not significant.)

HuD, a member of the Hu family of RBPs, regulates the translational rate and mRNA stability of its target genes by directly binding to their mRNA (Jung and Lee 2021 apr 23). Given the reduced FAO rate observed in βTC6-shHuD cells, we reasoned that HuD modulates the expression of genes involved in mitochondrial FAO (Figure 2A). To test this, we first performed RNA immunoprecipitation (RIP) using an anti-HuD antibody. Comparative analysis of FAO-related mRNA levels revealed a significant enrichment of LCAD mRNA in HuD-containing RNP complexes (Figure 2B, Supplementary Figure S1B), suggesting that HuD may regulate mitochondrial FAO by modulating LCAD expression. To further evaluate the regulation of LCAD expression by HuD, we analyzed both mRNA and protein levels of four ACAD genes in βTC6 cells. As shown in Figure 2C, HuD knockdown via short interfering RNA (siRNA) did not alter the mRNA levels of ACAD genes compared to control cells (Figure 2C), indicating that HuD does not influence their mRNA turnover. Importantly, HuD depletion significantly reduced LCAD protein levels in βTC6 cells, whereas the expression of other ACADs remained unchanged (Figure 2D). Similarly, βTC6-shHuD cells exhibited a propounded reduction in LCAD protein expression (Figure 2E). In contrast, HuD overexpression selectively increased LCAD protein levels without affecting other ACADs (Figure 2F). These results suggest that HuD serves as a critical regulator of LCAD expression at the post-transcriptional level in βTC6 cells.

### Hud regulates LCAD expression by interacting with its 3'UTR

**Figure 3.**
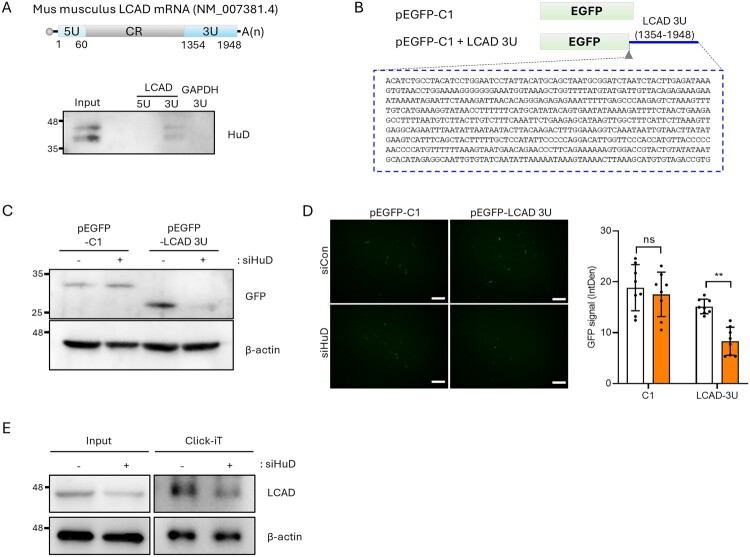
HuD regulates LCAD expression by interacting with the LCAD 3’UTR. (A) Left: Schematic diagram of *Mus musculus* LCAD mRNA (NM_007381.4). The untranslated regions of LCAD mRNA were *in vitro* transcribed into biotinylated RNA probes. Right: Biotinylated RNA probes (LCAD 5U, 3U, and GAPDH 3U) were incubated with lysates prepared from βTC6 cells and probe-interacting proteins were pulled down using streptavidin-coupled magnetic beads. The interaction between the UTRs of LCAD and HuD was examined by Western blotting using an anti-HuD antibody. Biotinylated GAPDH 3U was used as a negative control. (B) Schematic diagram of EGFP reporter incorporating the LCAD 3'UTR (1354-1948 nt, pEGFP-LCAD 3U) and control vector (EGFP-C1) (▴: stop codon). (C, D) Cells were sequentially transfected with siRNA and the reporter plasmid followed by analysis of relative GFP expression by Western blotting (C) and quantification of relative fluorescence intensity using ImageJ (D). (E) De novo synthesized LCAD protein was assessed using the Click-iT™ system, followed by biotin enrichment and Western blotting with an anti-LCAD antibody. Scale bar represents 200 μm. β-actin was used as a loading control, and data are expressed as mean ± SD from each image. Statistical significance was determined using Student’s t-test (**p < 0.01; ns, not significant).

Given our observation that LCAD is reduced in HuD knockdown cells and that the HuD-containing RNP complexes specifically binds to LCAD mRNA, we next investigated the underlying regulatory mechanisms. RBPs often modulate gene expression by interacting with untranslated regions (UTRs) of target mRNAs. To assess the association between HuD and the UTRs of LCAD mRNA, we performed RNA pull-down assays using biotinylated fragments corresponding to the 5’UTR and 3’UTR of LCAD mRNA. Following incubation with cell lysates, HuD binding to these RNA fragments was evaluated by immunoblotting with an anti-HuD antibody. Notably, our results revealed that HuD selectively interacts with the 3’UTR of LCAD mRNA (Figure 3A). Next, to determine whether HuD regulates LCAD expression through this interaction, we constructed an enhanced green fluorescent protein (EGFP) reporter construct containing the LCAD 3’UTR sequence (1351–1948 nt; pEGFP-LCAD 3U) positioned downstream of the EGFP coding sequence (Figure 3B). Indeed, depletion of HuD markedly reduced EGFP protein expression in EGFP-LCAD 3U reporter transfected βTC6 cells, whereas no significant changes were observed in cells transfected with the control reporter (EGFP-C1) (Figure 3C). Consistently, a decrease in fluorescence intensity was also observed in HuD knockdown cells (Figure 3D). Finally, to determine whether HuD regulates the translation of LCAD mRNA, de novo protein synthesis was assessed using the Click iT™ system. We observed that HuD knockdown significantly reduced the levels of newly synthesized LCAD protein (Figure 3E). Collectively, these results indicate that HuD enhances LCAD translation by directly interacting with its 3’UTR.

### Depletion of HuD sensitized cells to lipotoxic stress

**Figure 4.**
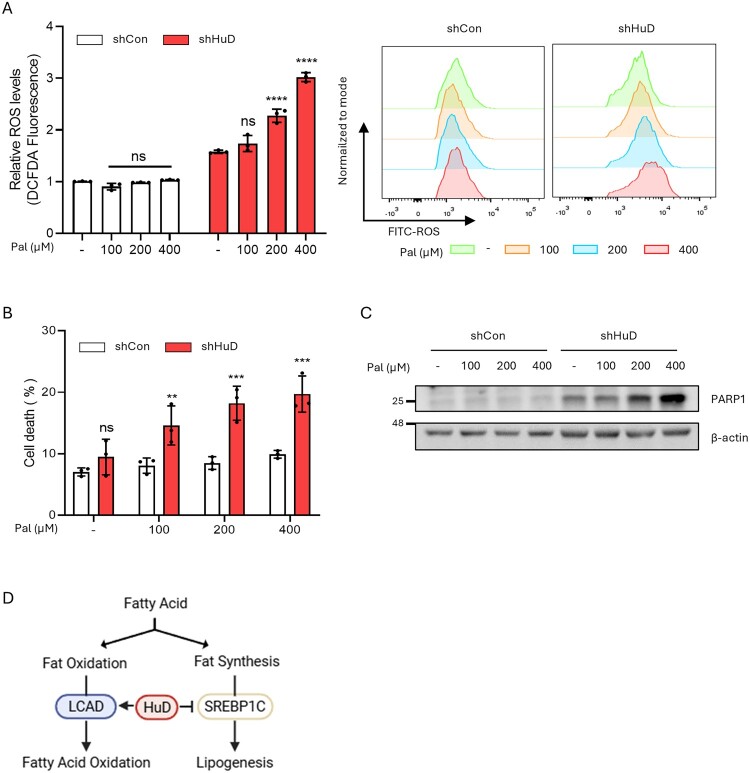
HuD downregulation sensitizes pancreatic β-cells to lipid-induced stress. (A) Relative ROS levels in shCon and shHuD βTC6 cells incubated with or without palmitate for 24 h. Statistical analysis was performed using two-way ANOVA with Dunnett’s multiple comparisons test. (B) Relative cell death in shCon and shHuD βTC6 cells treated with or without palmitate. Statistical significance was determined using a two-way ANOVA with Sidak’s multiple comparisons test. (C) PARP1 protein levels in whole-cell lysates from βTC6 cells incubated with or without palmitate for 24 h. (D) Schematic representation of HuD-mediated regulation of fatty acid oxidation and lipid biosynthesis. Representative data are presented as mean ± SD from each image. (**p < 0.01; ***p < 0.001;****p < 0.0001; ns, not significant.)

Excess lipid accumulation induces lipotoxicity, a condition characterized by cellular dysfunction and damage (Oh et al. 2018). A key driver of lipotoxicity is oxidative stress, primarily caused by excessive ROS production (Ly et al. 2017; Lipke et al. 2022). It has been reported that impaired FAO inducing cellular oxidative stress by increasing ROS generation through mitochondrial electron leakage and alternative oxidation pathways (Serra et al. 2013). Given the lipid accumulation and reduced FAO observed in HuD-depleted βTC6 cells, we reasoned that HuD knockdown increases ROS production, inducing oxidative stress and enhancing susceptibility to death under lipid-overloaded conditions. To test this hypothesis, we examined whether HuD directly modulates ROS production in response to palmitate supplementation. Importantly, palmitate treatment increased cellular ROS levels in a dose-dependent manner in HuD knockdown cells, while having no effect on ROS levels in control cells (Figure 4A). Next, to assess the functional significance of HuD-mediated FAO regulation, we examined its impact on cellular sensitivity to palmitate-induced lipotoxicity. In line with the increased ROS production, HuD knockdown rendered cells more sensitive to palmitate treatment compared to control cells (Figure 4B). This increased sensitivity was accompanied by elevated levels of cleaved PARP1, a key marker of apoptosis, indicating enhanced apoptotic cell death (Figure 4C). Taken together, our results suggest that HuD plays a crucial role in maintaining cellular fatty acid homeostasis, and its depletion exacerbates lipotoxicity in βTC6 cells under lipid-stressed conditions.

## Discussion

Here, we identified the RNA-binding protein HuD as a key regulator of mitochondrial FAO and lipid homeostasis in βTC6 cells. HuD knockdown led to significant lipid accumulation, exacerbated by palmitate treatment, and impaired FAO. Mechanistically, HuD directly regulated LCAD expression by binding to its 3’UTR, enhancing its translation without affecting mRNA levels. Additionally, HuD depletion increased ROS production in response to palmitate treatment, inducing oxidative stress and apoptosis. These findings underscore the critical role of HuD in regulating mitochondrial FAO, and its loss disrupts fatty acid homeostasis, leading to oxidative stress and lipotoxic cell death in pancreatic β-cells.

We observed that HuD knockdown leads to lipid accumulation and impaired FAO in pancreatic cells. Excess lipid deposition and dysregulated FAO have been associated with cellular stress and dysfunction across multiple tissues (Geng et al. 2021; Tian et al. 2023; Sun et al. 2025). In pancreatic islets, prolonged lipid accumulation and lipotoxicity have been implicated in β-cell dysfunction and may contribute to islet inflammation or fibrosis (Geng et al. 2021). Additionally, FAO defects can lead to energy imbalance and mitochondrial dysfunction, which may further exacerbate pathological conditions (Hwang and Chung 2021). Thus, the metabolic disruptions observed following HuD depletion raise the possibility that HuD may play a role in maintaining islet health by regulating lipid homeostasis. Future studies are needed to determine whether HuD also influences fibrotic or inflammatory pathways in the pancreas.

Our studies highlight a critical role HuD in regulating mitochondrial FAO, primarily through its control of LCAD expression. While our findings focus on the HuD-LCAD axis, it is possible that HuD also influences FAO through additional mechanisms. RNA-binding proteins often regulate multiple metabolic pathways. Indeed, HuD has been previously shown to modulate the expression of Insig1, which is involved in lipid metabolism (Kim et al. 2016). Thus, HuD may also affect the expression or activity of other FAO-related enzymes or influence mitochondrial biogenesis via upstream regulators such as peroxisome proliferator-activated receptor gamma coactivator-1 alpha (PGC-1α). Similarly, HuD might influence FAO by regulating lipid transport proteins or transcription factors like peroxisome proliferator-activated receptor alpha (PPARα), a key driver of FAO-related gene expression. Notably, HuR, another member of the ELAV-like protein family, has been shown to regulate lipid metabolism by modulating mRNA levels of genes involved in fatty acid metabolism, with its depletion resulting in reduced lipid oxidation in muscle cells (Mynatt et al. 2019). Given the shared RNA-binding specificity between HuD and HuR, it will be important to explore whether these Hu proteins coordinately regulate cellular fatty acid homeostasis, potentially through target switching or compensatory mechanisms. Exploring these possibilities may reveal new insights into the role of HuD in lipid homeostasis and β-cell function.

Our findings align with a previous study linking HuD to triacylglycerol synthesis, suggesting that HuD facilitates fatty acid utilization as an energy source by redirecting fatty acids away from storage toward mitochondrial oxidation (Figure 4D). Interestingly, HuD also represses insulin secretion by inhibiting its translation (Lee et al. 2012). Since insulin promotes lipid storage while inhibiting fatty acid utilization at the whole-body level, HuD may function as a key regulator of fatty acid metabolism both within cells and systemically, through direct and indirect mechanisms.

These findings not only uncover a previously unrecognized role of HuD in the regulation of FAO but also suggest broader physiological significance. Impaired FAO and lipid accumulation are key features of metabolic diseases such as type 2 diabetes and nonalcoholic fatty liver disease. By maintaining FAO capacity and preventing lipotoxic stress, HuD may contribute to the preservation of β-cells function under metabolic stress conditions. Therefore, targeting HuD or its downstream pathways could hold therapeutic potential for restoring metabolic homeostasis and protecting β-cell function against lipotoxicity.

In conclusion, our findings reveal a novel role for HuD in mitochondrial FAO. We have demonstrated that HuD knockdown impairs FAO by reducing LCAD expression in pancreatic β-cells. This function is particularly critical under lipid-stressed conditions, where HuD depletion leads to increased ROS production and lipotoxic cell death. These findings highlight HuD as a potential therapeutic target for preserving pancreatic β-cells function and regulating cellular fatty acid homeostasis.

## Supplementary Material

Supplementary Material
