# Supplementary material for: RNA binding protein HuD regulates fatty acid oxidation in pancreatic β-cells by modulating long-chain acyl-CoA dehydrogenase expression
Source: Anim Cells Syst (Seoul). 2025 Aug 11;29(1):512–22. doi: 10.1080/19768354.2025.2542168 (PMC12340947; doi:10.1080/19768354.2025.2542168)
Supplement: Supplementary Material [file TACS_A_2542168_SM4138.docx]

**RNA binding protein HuD regulates fatty acid oxidation in pancreatic β-cells by modulating long-chain acyl-CoA dehydrogenase expression**

**Supplemental Figure 1**


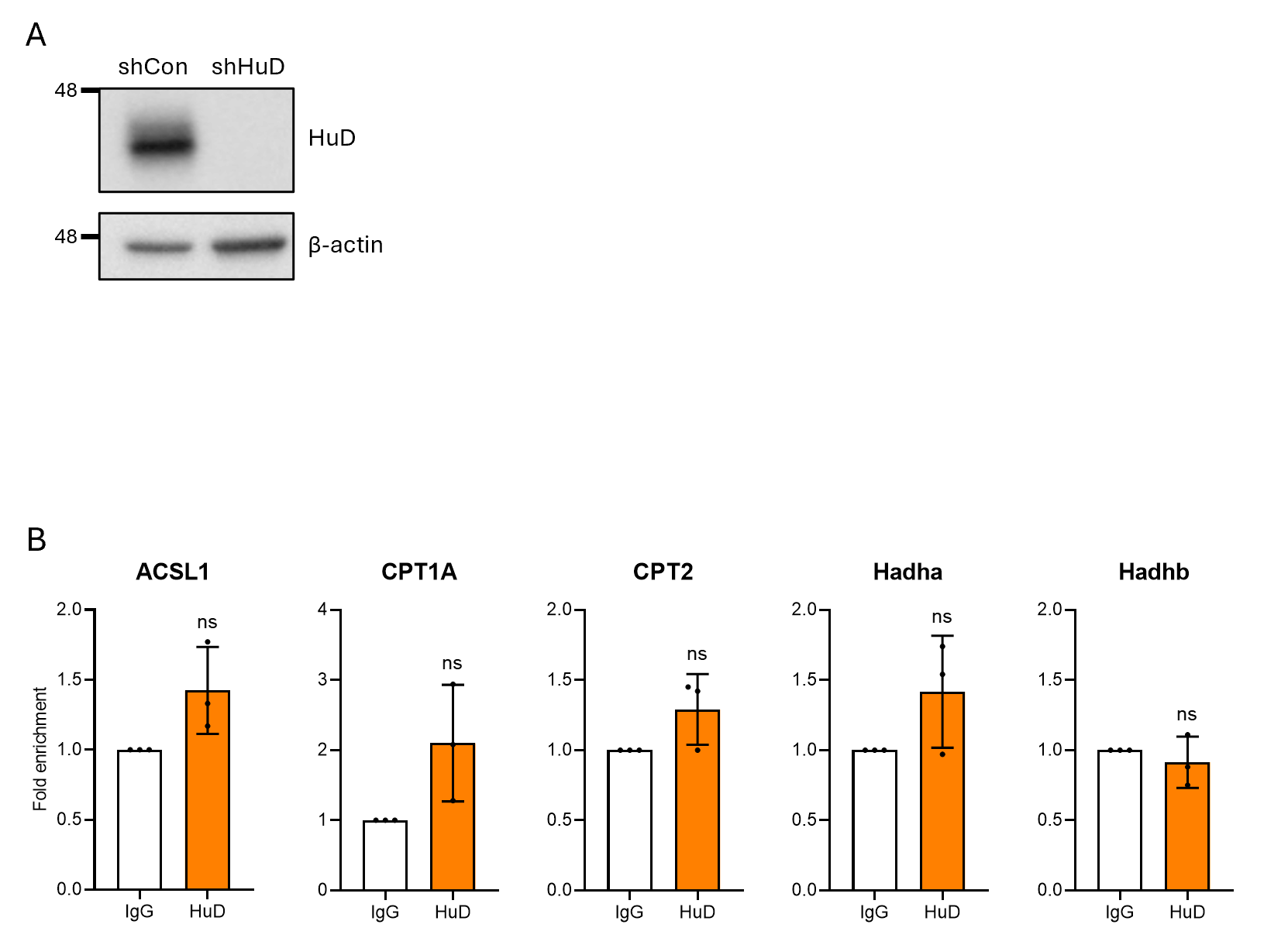


(A) HuD expression was confirmed by Western blotting with β-actin as a loading control. (B) Interaction between HuD and putative target mRNAs involved in fatty acid oxidation (FAO) was analyzed by RNA immunoprecipitation using HuD or control IgG antibodies, followed by RT-qPCR. Statistical significance was determined using Student`s t-test.
